# Indications and Relative Utility of Lower Endoscopy in the Management of *Clostridium difficile* Infection

**DOI:** 10.1155/2011/626582

**Published:** 2011-10-17

**Authors:** Nora E. Burkart, Mary R. Kwaan, Christopher Shepela, Robert D. Madoff, Yan Wang, David A. Rothenberger, Genevieve B. Melton

**Affiliations:** ^1^Division of Colon and Rectal Surgery, Department of Surgery, University of Minnesota, 420 Delaware Street SE, Mayo Mail Code 450, Minneapolis, MN 55455, USA; ^2^Institute for Health Informatics, Academic Health Center, University of Minnesota, Minneapolis, MN 55455, USA; ^3^Division of Gastroenterology, Department of Medicine, University of Minnesota, Minneapolis, MN 55455, USA

## Abstract

*Background*. Diagnosis and management of *Clostridium difficile* infection (CDI) rely upon clinical assessments and diagnostic studies. Among diagnostic tests, lower gastrointestinal (GI) endoscopy in the setting of CDI remains controversial. *Objective*. To describe the role of lower endoscopy in CDI management. *Methods*. Retrospective study of lower endoscopies in CDI at four metropolitan hospitals, July 2005 through December 2007. *Results*. Of 1760 CDI inpatients, 45 lower endoscopies were performed on 43 patients. Most common indications were ruling out other etiologies (42%), inconclusive stool studies (36%), and worsening course (11%). Most endoscopies (73%) had positive findings, including pseudomembranous colitis (49%) and nonspecific colitis (24%). Biopsies were performed in 31 cases, more with nonspecific colitis (10/11, 92%) compared to pseudomembranous colitis (14/22, 64%). *Conclusion*. While not recommended as a primary screening tool, lower GI endoscopy can add valuable information in CDI when other colonic pathologies may exist, studies are inconclusive, or clinical status worsens.

## 1. Introduction

*Clostridium difficile* infection (CDI) is a significant public health problem due to its association with antibiotic use and healthcare settings, increasing overall incidence, evolving epidemiology, and high associated health care costs. For any individual patient with symptomatic CDI, the spectrum of disease can vary widely. While initial treatment is effective in most cases, some cases are highly complex including patients with recalcitrant or recurrent disease [1], infections caused by increasingly virulent strains of *Clostridium difficile (C. difficile) *that are unresponsive to traditional medical therapy [2–4], and patients with fulminant colitis requiring surgery [5–10]. 

Stool studies like enzyme immunoassays for *C. difficile* toxins [3, 11] have improved our diagnostic abilities with CDI. The diagnosis, management, and treatment of CDI, particularly in the acute inpatient setting, rely upon a combination of ongoing clinical assessments and diagnostic studies. Lower gastrointestinal (GI) endoscopy, either flexible sigmoidoscopy or colonoscopy, can be used to (1) visualize the colon looking for inflammation or for the presence of pseudomembranes and (2) to obtain tissue and stool for diagnostic purposes. 

The optimal role of the lower GI endoscopy in the setting of CDI remains poorly defined and controversial. In the late 1970s and 1980s, some authors stressed the importance of endoscopy as a diagnostic tool [12, 13]. In the 1990s, with the improvement in the speed and reliability of the microbiologic diagnostic tests, it was thought to be less useful due to the cost and the relative lack of sensitivity of endoscopy as a general screening tool. As such, lower GI endoscopy began to play a secondary role in the workup of antibiotic-associated diarrhea [14]. 

Over the past decade, the pattern of CDI has changed and evolved. CDI has become a more frequent and severe disease that is increasingly refractory to standard therapy and likely to relapse [2, 3, 15]. This more severe version of the disease has been associated with the use of relatively new antibiotics like fluoroquinolones [2–4, 16, 17] and a new strain of *C. difficile* known as BI/NAP1/ribotype 027, which was first reported during outbreaks in Quebec, Canada in the early 2000s [16]. There is only one study that has looked at the role of lower GI endoscopy during this later epidemiological presentation [18], and the authors concluded that lower GI endoscopy was superior to the exclusive use of *C. difficile* cytotoxin testing of stools in the subgroup of patients with pseudomembranous colitis and that flexible endoscopy should be considered in all hospitalized patients with diarrhea in whom the stool test for *C. difficile* cytotoxin and enteric pathogens is negative but a high level of clinical suspicion remains.

The goal of this paper is to describe the use and role of flexible lower GI endoscopy with CDI in an inpatient setting including exact indication for the study, utility of the endoscopy, histopathological findings, and outcomes. 

## 2. Methods

Data were collected from four metropolitan hospitals (Fairview Health Services in Minnesota, USA), including the University of Minnesota Medical Center (July 2005 through December 2007), on all inpatients with a documented diagnosis of CDI (ICD-9CM code 008.45) and a lower GI endoscopy (ICD-9 procedure codes for sigmoidoscopy (45.24) or colonoscopy (45.22, 45.23, 45.25, 45.42, 45.43)) during the same admission. We included patients who underwent lower GI endoscopy for an indication related to assisting with the diagnosis, followup, or treatment of CDI. Those patients with CDI who had endoscopy performed for other indications like rectal bleeding or chronic anemia were excluded as were patients with a known history of inflammatory bowel disease. Demographic data, patient comorbidities, and known risk factors for the development of CDI were recorded including current or recent hospitalization, age older than 65 years, recent surgery, and antibiotic exposure. History of antibiotic exposure including class of antibiotic, previous episodes of CDI, the presence or absence of diarrhea, and characteristics of the diarrhea was also included as baseline data. 

CDI was considered “hospital onset” if the patient developed symptoms as an inpatient and “community onset” if the patient was admitted to the hospital with CDI symptoms that started in the community. Patients were also classified as immunosuppressed if the patient had one or more of the following: chronic systemic corticosteroids, cancer, HIV/AIDS, or organ transplant. Clinical status at the time of endoscopy was also recorded including the presence of fever, leukocytosis, albumin level, presence of septic shock, and admission to the intensive care unit. 

Patient records were carefully reviewed to ascertain how or if the diagnosis of CDI had been established at the time of lower GI endoscopy, including clinical diagnosis, outcomes of stool assays (*C. difficile* toxins A/B, *C. difficile* toxin A, and *C. difficile* culture), and use of imaging studies (particularly use of abdominal CT scan). With respect to lower GI endoscopy, the specific clinical indication was determined and classified into categories: ruling out other etiologies, high clinical suspicion despite inconclusive stools studies (negative or pending), worsening course of disease, and therapeutic reasons (e.g., bowel decompression, placement of rectal tube, medication enemas). Lower GI endoscopy was considered to have been performed to rule out other etiologies when the CDI diagnosis was already established (clinical suspicion and *C. difficile* toxin A or A/B (+), or *C. difficile* culture (+)) but there was the need to rule out other etiologies due to other clinical factors where additional colonic pathology could be occurring simultaneously, including persistent symptoms, bloody diarrhea or immunosuppression (risk of *Cytomegalovirus* (CMV) colitis and/or graft versus host disease (GVHD) specially in transplant patients).

Endoscopic findings, biopsy results, initial therapy, and postprocedure therapy after performing endoscopy and response to treatment were also collected. Antibiotic therapy with metronidazole orally or intravenously, vancomycin orally or with enemas, and/or rifaximin orally was also determined. Surgery as initial or later therapy and recurrence/relapse of the disease were also recorded. 

Comparisons between groups of patients were made using analysis of variance, chi-square statistics, Fisher exact test, and Student's *t-*test as appropriate. Significance was accepted at the 5% level. University of Minnesota Institutional Review Board approval was obtained and informed consent waived for this study.

## 3. Results

### 3.1. Patient Demographics, Comorbidities, Risk Factors, and Clinical Presentation

Over the two-and-a-half-year period, there were 1760 total cases of inpatient CDI of which 43 inpatients (mean age 59, 53% female) underwent 45 total lower GI endoscopy studies to assist with the diagnosis and/or management of CDI. A summary of patient characteristics, comorbidities, risk factors, and clinical presentation is included in Table 1. The most important comorbidities were cardiac disease (65%), immunosuppression (56%), pulmonary disease (40%), diabetes (26%), and chronic renal failure (26%). The use of steroids (49%) was the most important cause for immunosuppression, followed by cancer (30%) and transplant (28%). Of 12 patients with transplants, 6 (50%) had bone marrow transplants, 3 (25%) lung transplants, and 3 (25%) kidney and/or pancreas transplant. Of note, HIV/AIDS was considered as one cause of immunosuppression, but we did not have any patient with this condition in our sample.

Most of the patients were found to have exposure to antibiotics (91%) before developing CDI. Fluoroquinolones were most often the inciting antibiotic (57%), followed by cephalosporins (13%). A history of recent surgery, GI or non-GI, was found in 15 patients (35%). There were 16 (37%) patients with recurrent disease, including 10 (62.5%) with persistent disease and 6 (37.5%) with delayed recurrence. 

While 36 patients (84%) had CDI which started prior to hospitalization, in the community, 29 (81%) had a history of a recent hospitalization before developing CDI. Seven patients (16%) were admitted to the hospital for other reasons and developed CDI during the course of their stay (hospital onset). While one patient presented with an ileus, all others had diarrhea at presentation. Fever was present in 18 (42%), abnormal white blood count in 29 (67%), albumin was less than 3.2 mg/dL in 20 (47%), septic shock in 7 (16%), and ICU admission in 8 (19%).

### 3.2. Use of Ancillary Studies

Overall, only 22 (51%) patients had positive *C. difficile* toxins A or A/B, with 13 of 22 (59%) with a positive *C. difficile* stool culture. From the 21 patients with negative *C. difficile* toxins A or A/B, 12 of 21 (57%) had a positive *C. difficile* stool culture. 

There were 9 patients with both negative *C. difficile* toxins A or A/B and *C. difficile* stool culture. In this subgroup of patients, the lower endoscopy was used as a diagnostic tool. Lower endoscopy was positive in 8/9 (88%) patients, with PMC in 6 cases and non-specific colitis in 2 cases. The biopsies of these 2 nonspecific colitis cases showed 1 PMC and 1 ischemic colitis. The only patient with both negative *C. difficile* toxins A or A/B and *C. difficile* stool culture that had negative lower endoscopy was a bone marrow transplant patient. This same patient had another endoscopy later in his hospital stay that showed PMC.

Of the 16 (37%) patients with recurrent disease there were 11 (69%) with negative culture or toxin stool tests, including 9 (56%) recurrent patients with a negative *C. difficile* toxin test (four with a negative culture) and two negative *C. difficile* culture results in the 7 (44%) recurrent patients with a positive *C. difficile *toxin test. In the 27 (63%) patients with a new diagnosis of CDI, 19 (70%) had one or more negative tests, including 12 (44%) patients with a negative *C. difficile* toxin test (5 with a negative stool culture) and seven negative *C. difficile* culture results in the 15 (56%) patients with a positive *C. difficile * toxin test. 

An abdominal CT scan was performed in 28 (65%) patients. CT scan findings were positive for colonic pathology in 25 (89%) patients, including pancolitis in 15 (54%) and segmental colitis in 10 (36%). Ascites was observed in 4 patients that had pancolitis. Free air was not found on any abdominal CT scan.

### 3.3. Indications of Lower GI Endoscopy

Of 45 lower GI endoscopic procedures, 28 (62%) flexible sigmoidoscopies and 17 (38%) colonoscopies were performed in 43 patients. Two patients had two endoscopies performed during the same hospital stay. Indications for these studies are summarized in Table 2. The most common indication was ruling out other colonic etiologies in 19 (42%) patients, including (a) four immunosuppressed patients where there was a need to rule out CMV colitis and/or GVHD, (b) four patients with bloody diarrhea where inflammatory bowel disease, ischemic colitis, or malignancy was suspected, and (c) eight patients with persistent diarrhea despite medical treatment for CDI. 

Another 16 (36%) patients underwent lower GI endoscopy due to inconclusive *C. difficile *studies, particularly when *C. difficile *toxins A and A/B were either negative or pending. Of these, 14 patients had a high clinical suspicion for CDI, but *C. difficile* toxins were negative and cultures pending at the time of lower GI endoscopy, and 2 patients had recurrent symptoms where lower GI endoscopy was performed prior to any stool study results due to a suspicious CT scan.

There were 5 (11%) patients who underwent lower endoscopy in the setting of a worsening clinical course including progression to septic shock or multisystem organ failure. Of these, two flexible scopes were performed following surgery (subtotal colectomy) for CDI to assess the rectal stump in the setting of multisystem organ failure. Another four (9%) lower endoscopies were indicated to followup on a known disease/condition, including two with a history of microscopic colitis and another two in the postoperative period following colectomy. One patient underwent lower GI endoscopy for bowel decompression (therapeutic) with the flexible endoscopy demonstrating pseudomembranous colitis.

### 3.4. Lower GI Endoscopy Findings and Pathology

Lower GI endoscopy demonstrated pseudomembranous colitis in 22 (49%) patients, nonspecific colitis or inflammation without pseudomembranes in 11 (24%), and normal mucosa in 12 (27%) patients (Table 3). There were no complications associated with lower GI endoscopy like perforations or major bleeding after biopsy. In the 31 biopsies performed, histology demonstrated pseudomembranous colitis in 13 (42%) and infectious colitis in 5 (16%). Biopsy was done less commonly when the endoscopy showed visible pseudomembranous colitis (14 of 22) than when it showed nonspecific colitis or inflammation without pseudomembranes (10 of 11), although this was not statistically significant (63.6% versus 90.9%, *P* = 0.09).

### 3.5. Outcomes following Lower GI Endoscopy

Table 4 summarizes alterations in CDI-directed therapy following lower GI endoscopy and patient outcomes. Initial therapy was metronidazole (oral or intravenous) in 29 patients, oral or enema administration of vancomycin in 4 patients, and vancomycin and metronidazole in 10 patients at the time of lower GI endoscopy. Following endoscopy, medical therapy was not changed in 27, escalated in 11, decreased in 2, and changed to surgical treatment in 3. Subtotal colectomy and ileostomy were performed in 3 patients with fulminant colitis. Two patients resolved CDI following surgery with no recurrent episodes. One patient following colectomy progressed to multisystem organ failure, followed by comfort care and death. CDI completely resolved in 28 out of 43 patients (65%); 11 (26%) patients had recurrent disease, and 4 (9%) patients progressed to multisystem organ failure and died after comfort care/withdrawal of support.

## 4. Discussion

The management of CDI, particularly in the hospitalized patient in the acute setting, relies upon a combination of ongoing clinical assessments and diagnostic studies. Among these tests, lower GI endoscopy, either flexible sigmoidoscopy or colonoscopy, can be used both to visualize the colon and to obtain tissue for diagnostic purposes. Its role in the management of CDI, however, has remained controversial and poorly defined. In this study we retrospectively analyzed inpatients with CDI who underwent lower GI endoscopy during the same admission to assist in the diagnosis, followup, or treatment of CDI. 

There are several limitations of this study, including its retrospective nature and coverage of only the inpatient setting. While our use of ICD-9 codes may slightly underestimate the use of lower GI endoscopy with CDI, previous studies have demonstrated that ICD-9 CM codes closely approximate true CDI, especially in symptomatic patients where test results are available at discharge and can be therefore used as a reasonable alternative to microbiological data for cohort identification [19]. Another limitation of this study is the use of enzyme toxin assays as the main initial diagnostic test. Currently, many health care institutions are moving to use PCR studies for CDI diagnosis. PCR studies have a much higher sensitivity, lower rate of false negative results, and faster results availability. This study was conducted prior the use of PCR as diagnostic test.

Forty-five lower GI endoscopy procedures were performed in 43 patients over two and half years most often to rule out additional colonic etiologies, to confirm CDI when *C. difficile* stool testing was inconclusive, and to confirm CDI and response to treatment in the setting of a worsening clinical course. Similar to previous reports [14, 20], pseudomembranous colitis was demonstrated in 22 endoscopies (49%), nonspecific colitis in 11 (24%), and normal mucosa in 12 (27%). 

Historically, in the late 1970s and 1980s, lower GI endoscopy use in the diagnosis of CDI was encouraged [13], as the etiological agent for CDI was not identified until 1978. In the 1990s, with the availability of the microbiological diagnostic tests, lower GI endoscopy was considered costly, invasive, and insensitive. It was relegated to a secondary role in the workup of antibiotic-associated diarrhea [14, 20]. More recently, CDI patterns have changed and evolved since the early 2000s, turning to a more frequent and severe disease that is increasingly refractory to standard therapy and more likely to relapse [2, 3, 15]. This more severe version of the disease is associated with the use of relatively new antibiotics like fluoroquinolones [2–4, 17, 21] and a new strain of *C. difficile* known as BI/NAP1/ribotype 027. One recent study analyzing the use of lower endoscopy with CDI [18] suggested that flexible sigmoidoscopy was superior to stool *C. difficile* cytotoxin testing in the subgroup of patients with pseudomembranous colitis. 

We found that the most common indication for lower GI endoscopy in CDI patients was the need to rule out coexisting pathology with CDI (42%) where the CDI diagnosis was already established prior to the performance of the lower GI endoscopy. This was particularly true in solid organ transplant and bone marrow transplant patients, where conditions like graft versus host disease or CMV colitis can occur simultaneously with CDI and require significantly different approach in treatment and management [22]. With solid organ transplant patients, CDI is one of the most common causes of diarrhea and has been increasing in incidence since the early 2000s [23]. Moreover, in certain populations, particularly lung transplant patients who are exposed regularly to antibiotics, CDI can progress into a life-threatening condition with fulminant colitis [24]. 

In addition, a subset of patients with CDI presented with concomitant bloody diarrhea which led to lower GI endoscopy to rule out other etiologies including ischemic colitis, inflammatory bowel disease, or severe pseudomembranous colitis. In patients with CDI and a high clinical suspicion for IBD, several etiologic processes may be occurring: an IBD flare with colonization by *C. difficile*, superimposed CDI without IBD flare, or both processes occurring simultaneously [25]. We did not observe a new diagnosis of IBD in this setting. Most of these cases (9/10) represented a severe presentation of CDI (with six having pseudomembranous colitis on endoscopy and three having nonspecific colitis). While there have been reports that patients on immunosuppressive treatments with CDI typically do not have pseudomembranes [26], we observed an immunosuppressed patient with bloody diarrhea with severe pseudomembranes.

We found that lower GI endoscopy was particularly helpful in confirming the diagnosis of CDI in patients with a high level of clinical suspicion for CDI but inconclusive stool tests (either negative or pending). This was consistent with the prospective findings of Johal et al. who concluded that, in this particular subset of patients, flexible sigmoidoscopy was superior to the stool *C. difficile* cytotoxin test [18]. This setting is one where other authors tend to agree on the use of lower endoscopy as an adjunctive tool for diagnosis of CDI [18, 27]. Overall in our sample study, only 22 (51%) patients had positive *C. difficile* toxins A or A/B. This partially could be attributed to the nature of our study sample, which only included patients with CDI that had an endoscopy. Since the sensitivity of *C. difficile* toxin A or toxins A/B tests of this era is 70%-80%, it requires repeat testing and if high clinical suspicion exists, the initiation of empiric treatment should be initiated [11]. Of the 21 patients with negative *C. difficile* toxins, 15 (71%) had a positive endoscopy, including PMC in 10 (48%) and nonspecific colitis/inflammation without pseudomembranes in 5 (24%). There were 9 patients with both negative *C. difficile* toxins A or A/B and *C. difficile* stool culture. In this subgroup of patients, the lower endoscopy was used as a diagnostic tool. Lower endoscopy was positive in 8/9 (88%) patients, with PMC in 6 cases and nonspecific colitis in 2 cases. The biopsies of these 2 non specific colitis cases showed 1 PMC and 1 ischemic colitis. The only patient with both negative *C. difficile* toxins A or A/B and *C. difficile* stool culture that had negative lower endoscopy was a bone marrow transplant patient. This same patient had another endoscopy later in his hospital stay that showed PMC. These results confirm the utility of lower endoscopy in this subgroup of patients.

As a final set of indications, lower GI endoscopy was particularly helpful in cases of worsening clinical course to confirm fulminant colitis (i.e., need for surgery) or to confirm the status of other colonic pathology. In our series, worsening clinical course was the indication for lower GI endoscopy in 5 (11%) of the patients, mostly as patients were progressing to septic shock or multisystem organ failure. This indication has particularly high utility in cases of fulminant colitis where adjunct studies may not be definitive and timely treatment is imperative [5, 20]. We also observed 4 (9%) patients who underwent lower endoscopy in the setting of known secondary colonic pathology as a study for ongoing clinical assessment in response to therapy.

## 5. Conclusion

In summary, endoscopic findings in our study correlated with the findings of other authors that have reported positive results in ~70% of CDI cases [11, 14, 20] including pseudomembranous colitis (~50–60%) and nonspecific colitis or inflammation without pseudomembranes. We observed that lower GI endoscopy in CDI, when indicated, plays a role not only as a diagnostic tool but also in the overall management of the disease. Thus, the most common indication of lower GI endoscopy in the setting of CDI was to rule out other colonic pathologies that may be coexisting with CDI diagnosis and inconclusive *C. difficile* studies being the second most common indication.

## Figures and Tables

**Table 1:** Patients demographics, comorbidities, risk factors, and clinical presentation.

	All patients (*n* = 43)	Recurrent disease (*n* = 16)	Primary disease (*n* = 27)
Sex, M/F	20/23	6/10	14/13
Age, mean, y	59	62	58
Length of stay, d	12	14	11

Comorbidities			
Cardiac disease	28 (65%)	12 (75%)	16 (59%)
Immunosuppression	24 (56%)	11 (69%)	13 (48%)
Steroids	21 (49%)	11 (69%)	10 (37%)
Cancer	13 (30%)	7 (44%)	6 (22%)
Transplant	12 (28%)	6 (38%)	6 (22%)
Pulmonary disease	17 (40%)	6 (38%)	11 (41%)
Diabetes	11 (26%)	7 (44%)	4 (15%)
Chronic renal failure	11 (26%)	5 (31%)	6 (22%)

Risk factors			
Antibiotic exposure	39 (91%)	13 (81%)	26 (96%)
Current/recent hosp.	36 (84%)	16 (100%)	20 (74%)
Age >65	17 (40%)	6 (38%)	11 (41%)
Recent surgery	15 (35%)	3 (19%)	12 (44%)

Presentation			
Community*	36 (84%)	15 (94%)	21 (78%)
Hospital*	7 (16%)	1 (6%)	6 (22%)
Ileus, distended abdomen	1 (2%)	1 (6%)	—
Diarrhea	42 (98%)	15 (94%)	27 (100%)
Presence of fever	18 (42%)	4 (25%)	14 (52%)
Septic shock	7 (16%)	3 (19%)	4 (15%)
ICU admission	8 (19%)	3 (19%)	5 (19%)
WBC >11.0 or <4.0	29 (67%)	10 (63%)	19 (70%)
Albumin <3.2	20 (47%)	9 (56%)	11 (41%)

*Onset of CDI: in the community, or in the hospital while inpatient.

**Table 2:** Lower GI endoscopy indications.

	Overall *N* = 45	Recurrent disease *N* = 17	Primary disease *N* = 28
Rule out other etiologies	19 (42%)	6	13
Immunosuppression	4 (9%)	1	3
Bloody diarrhea	4 (9%)	—	4
Ongoing symptoms despite therapy	8 (18%)	4	4
Other	3 (7%)	1	2
*C. difficile* studies inconclusive	16 (35%)	8	8
Worsening clinical status	5 (11%)	2	3
Followup of known colonic pathology*	4 (9%)*	1	3
Therapy^+^	1 (2%)	—	1

*History of microscopic colitis (*n* = 2), followup of colonic surgery (*n* = 2). ^+^Bowel decompression.

**Table 3:** Lower GI endoscopy findings and histopathologic results on biopsy.

Endoscopy Findings		Histopathological results					
	Overall *N* = 45	Biopsy *N* = 31	PMC *N* = 13	Infect. colitis *N* = 5	Indeterminate or other colitis *N* = 7	Other *N* = 2*	Normal *N* = 4
PMC	22 (49%)	14	12	1	—	1	—
NSC	11 (24%)	10	1	4	5	—	—
Negative	12 (27%)	7	—	—	2	1	4

PMC: Pseudomembranous colitis. NSC: Nondiagnostic or indeterminate nonspecific colitis or inflammation without pseudomembranes. Negative: normal mucosa or no inflammation.

*One patient with concurrent CMV colitis (biopsy)/CDI had showed PMC at endoscopy. The other patient with negative endoscopy had GVHD at biopsy.

**Table 4:** Treatment: initial therapy, course/followup, overall outcome.

	Initial therapy			
	Overall *N* = 43*	Metronidazole *N* = 29	Vancomycin *N* = 4	Vanco + metronidazole *N* = 10
Treatment following lower GI endoscopy				
No change in therapy*	27 (60%)	18	4	5
Escalating medical therapy	11 (26%)	10	—	1
Decreasing medical therapy	2 (5%)	—	—	2
Surgery^+^	3 (7%)	1	—	2

Outcome				
Resolved	28 (65%)	20	2	6
Recurrent disease	11 (26%)	9	1	1
Death	4 (9%)	—	1	3

*One patient in the no change in therapy group was on oral vancomycin (allergic to metronidazole) with bone marrow transplant and *C. difficile*, CMV, and graft versus host disease. The first endoscopy demonstrated a negative biopsy, and there was no change in therapy. The patient did not improve, and a second endoscopy was performed demonstrating PMC and on biopsy CMV colitis. He progressed to multisystem organ failure and died.

^+^One patient in the surgery category had two endoscopies: one endoscopy prior to surgery and one endoscopy after surgery to assess the ileostomy and mucus fistulotomy.
